# Analysis of CMTM6 and CMTM4 expression as potential regulators of the PD-L1 protein and its association with prognosis in glioma cancer

**DOI:** 10.3724/abbs.2022108

**Published:** 2022-08-15

**Authors:** Hui Xue, Bin Qiu, Hao Wang, Ping Jiang, Weiwei Zhang, Lixiang Xue, Junjie Wang

**Affiliations:** Department of Radiation Oncology Peking University Third Hospital Beijing 100191 China

Chemokine-like factor super family (CKLFSF) is a gene family reported in 2003. In humans, the family is composed of nine members, namely chemokine-like factor (CKLF) and CKLF-like MARVEL transmembrane domain-containing member 1–8 (CMTM1–8) [1]. Genes in the CMTM family have different spliceosomes, which code for MAL-related proteins for vesicle trafficking and membrane link domain (MARVEL). Studies have shown that molecules containing this domain play important physiological and pathological roles in protein transport [2]. CMTM family plays an important role in the immune system and occurrence of tumors. Among them, CMTM3 and CMTM7 have functional characteristics of tumor suppressors, which are co-located with early endosomal marker Rab5, regulating the traffic and stability of membrane molecules, such as EGFR, VE-cadherin and BCR. Australian and Dutch researchers found that *CMTM6* is the only gene that can regulate PD-L1 expression in the absence of IFNγ. CMTM6 inhibits PD-L1 ubiquitination and extends its half-life, avoiding lysosome-mediated degradation. Inhibition of CMTM6 expression could significantly promote T cell activation. In CMTM6-knockout cells, CMTM4 also has the function of stabilizing PD-L1 and is the standby regulatory molecule of PD-L1 [ 3, 4] . These studies indicate that CMTM family plays an important role in the regulation of key membrane molecules involved in tumor genesis and development. CMTM6 and CMTM4 are new molecules that can be used to enhance the therapeutic benefits of immune checkpoint inhibitors. Both of them are prognostic biomarkers in several kinds of tumors, which have attracted increasing attention.

Gliomas are deadly and immunosuppressive brain tumor. Despite advances in comprehensive therapy, patients who suffer from gliomas still have a short median survival time due to the resistance to treatments and recurrence [5]. In the past few years, studies on anticancer immune therapies have promoted improvements to the limited success of conventional therapies. Antibodies targeting PD-L1 represent promising immunotherapies. Clinical trials of anti-PD-L1 drugs in glioma have been initiated. However, the rates of objective response and complete response are very low due to inadequate T- cell infiltration and immunosuppressive microenvironment [6]. Hence, new immune-related therapeutic targets have to be further exploited. Thus, in this study we evaluated the expressions of CMTM6 and CMTM4 in human glioma samples to assess its association with prognosis.

The upregulation/downregulation of CMTM6/CMTM4 in gliomas is supported by the TCGA ( http://ualcan.path.uab.edu) and CGGA ( http://www.cgga.org.cn) databases. CMTM6 mRNA level is significant higher while CMTM4 mRNA level is sharply lower in glioma than in normal tissue ( Supplementary Figure S1A,D). CMTM6 mRNA level is increased while CMTM4 mRNA level is decreased as pathological grades increase ( Supplementary Figure S1B,C), which indicates glioma prognosis. In addition, CMTM6/CMTM4 mRNA levels are significantly different in different molecular phenotypes of IDH ( Supplementary Figure S1E,F), which is closely related to the clinical prognosis and clinical treatment selection. These bioinformatics data suggest that CMTM6 and CMTM4 likely play an important role in gliomas.

We further detected the expressions of CMTM6/CMTM4 and PD-L1 in the same glioma tissue samples by multiplexed immunofluorescence staining assay. Tumor-associated macrophages (TAMs) were obtained from Shanghai Outdo Biotech (Shanghai, China), which contained 177 tumors, and the clinical characteristics of the patients are summarized in Supplementary Table S1 . All experimental procedures followed the Human Ethical Committee protocol (YB M-05-02). The results showed that CMTM6 and PD-L1 are located mainly on the cell membrane and in the cytoplasm, while CMTM4 is observed on the cell membrane, in the cytoplasm and nucleus ( Figure 1A). CD68 is located mainly on the cell membrane and in cytoplasm of macrophages, CD8 is located mainly on the cell membrane and in the cytoplasm of T cells, while Ki67 is observed mainly in the nucleus of tumor cells. CMTM6 and CMTM4 are detected in about 88% and 89.5% of samples, respectively. PD-L1 is detected in about 76.5% of samples.

**Figure 1:**
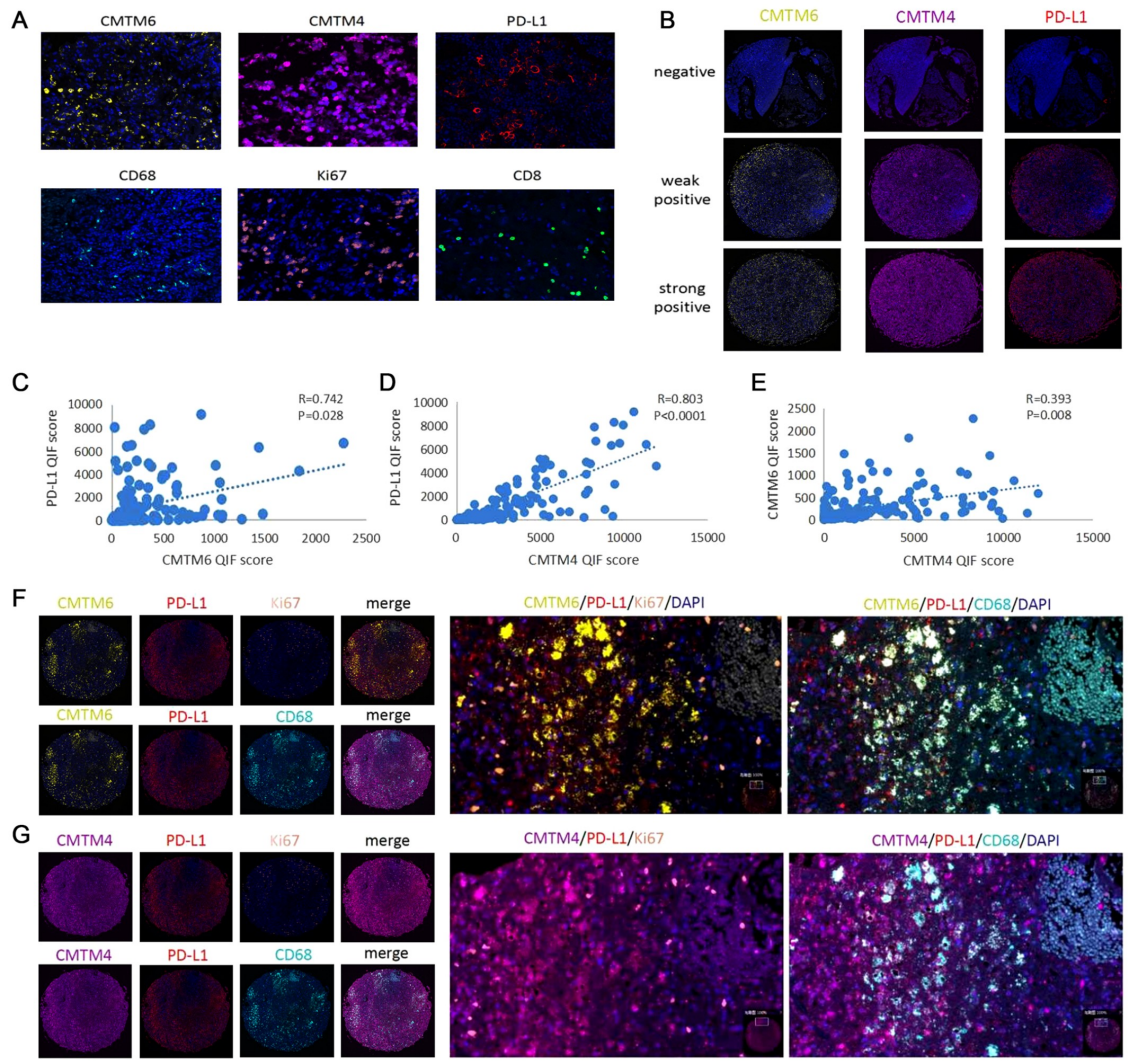
Figure 1 Correlation analysis of expressions of CMTM6/CMTM4/PD-L1 in glioma tissues (A) The localization of CMTM6/CMTM4/PD-L1/CD68/Ki67/CD8 staining in glioma tissues. CMTM6 and PD-L1 are located mainly on the cell membrane and in the cytoplasm, while CMTM4 is observed on the cell membrane, in the cytoplasm and nucleus. CD68 is located mainly on the cell membrane and in the cytoplasm of macrophages. CD8 is located mainly on the cell membrane and in the cytoplasm of T cells. Ki67 is observed mainly in the cell nucleus of tumor cells. (B) Negative, weak positive, and strong positive expressions of CMTM6/CMTM4/PD-L1. (C) Pearson correlation analysis of CMTM6 and PD-L1. (D) Pearson correlation analysis of CMTM4 and PD-L1. (E) Pearson correlation analysis of CMTM6 and CMTM4. (F) Expressions of CMTM6 and PD-L1 in Ki67+tumor cells and in CD68+macrophages. (G) Expressions of CMTM4 and PD-L1 in Ki67+tumor cells and in CD68+macrophages.

To verify the results of bioinformatics analysis, the expression of CMTM4/CMTM6/PD-L1 among different pathological grades of glioma was calculated after scoring the QIF in each sample. CMTM6, CMTM4 and PD-L1 protein levels are all increased as pathological grades increase ( Supplementary Figure S1G,I), which is corresponding to the bioinformatics analysis for CMTM6 and PD-L1 at the mRNA level. However, the CMTM4 protein level is not consistent with results of mRNA level from bioinformatics analysis, which needs more exploration. Furthermore, macrophages are major cells co-expressing CMTM6/CMTM4 and PD-L1, and co-localization of CMTM6/CMTM4 and PD-L1 is significantly higher in CD68 ^+^ macrophages than in Ki67 ^+^ tumors ( Figure 1F,G).

To confirm that CMTM6 and CMTM4 play a key role in maintaining the stability of PD-L1 [3], we analyzed the linear correlation of CMTM6/CMTM4 and PD-L1 by Pearson’s correlation assessment. Results showed that CMTM6 and CMTM4 expressions are significantly related to PD-L1 positivity ( Figure 1B–D). Thus, preventing CMTM6/CMTM4 from binding with PD-L1 may recover immunosuppression response and serve as a promising strategy for immunotherapy in glioma cancer.

We further analyzed whether the survival probability is related to CMTM6/CMTM4/PD-L1 protein level by Kaplan-Meier survival analysis and log-rank test. The median was used as the cut-off point to divide patients into high and low expression groups. Results showed that patients with high CMTM6/CMTM4/PD-L1 expression ( Figure 2A–C), high CMTM6/CMTM4 and PD-L1 co-expression ( Figure 2D,E) are associated with the lower OS compared to the patients with low CMTM6/CMTM4/PD-L1 expression, low CMTM6/CMTM4 and PD-L1 co-expression. However, OS is not significantly higher in patients with high CMTM6, CMTM4, or PD-L1 single expression in macrophages ( Figure 2F–H). Surprisingly, OS is significantly higher when CMTM6/CMTM4 and PD-L1 levels are high in the macrophages at the same time ( Figure 2I,J).

**Figure 2:**
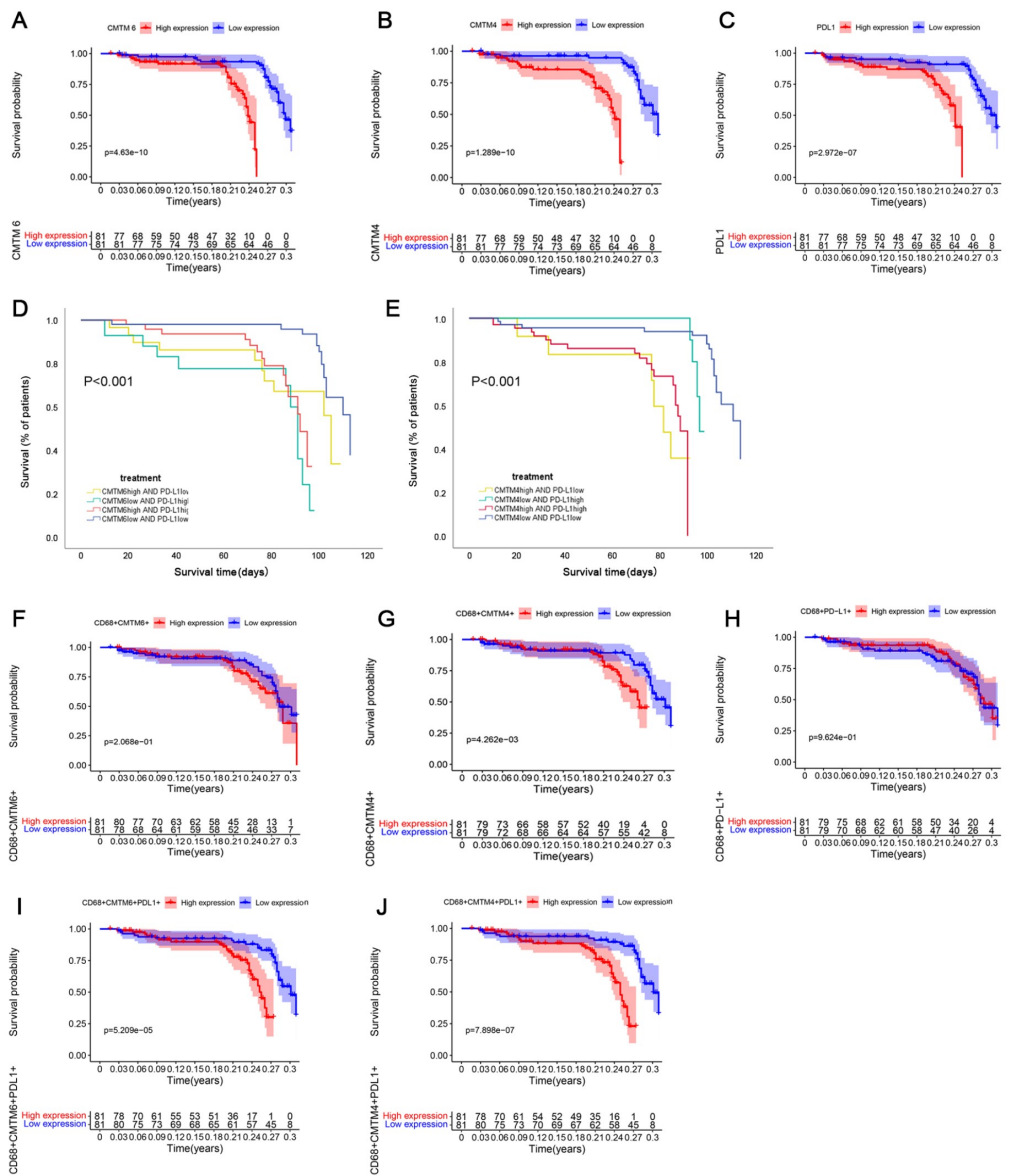
Figure 2 The overall survival of patients with gliomas (A) Patients with high CMTM6 expression ( n=81) showed a significantly lower overall survival than patients with low CMTM6 expression ( n=81). (B) Patients with high CMTM4 expression ( n=81) showed a significantly lower overall survival than patients with low CMTM4 expression ( n=81). (C) Patients with high PD-L1 expression ( n=81) showed a significantly lower overall survival than patients with low PD-L1 expression ( n=81). (D). Patients with high CMTM6 and high PD-L1 co-expression showed a significantly lower overall survival than patients with low CMTM6 and low PD-L1 co-expression. (E). Patients with high CMTM4 and high PD-L1 co-expression showed a significantly lower overall survival than patients with low CMTM4 and low PD-L1 co-expression. (F–H). Overall survival is not obviously extended in patients with high CMTM6/CMTM4/PD-L1 single expression in macrophages. (I–J). Overall survival is significantly higher in patients with CMTM6/CMTM4 and PD-L1 high co-expression in macrophages. Red represents high CMTM6 expression, and blue represents low CMTM6 expression.

Clinical factors associated with survival were analyzed by univariate and multivariate cox proportional hazard regression analysis. Results showed that CMTM6/CMTM4/PD-L1 expression is a risk factor for prognosis, however, CMTM6/CMTM4/PD-L1 is not an independent risk factor. In addition, grade is also an independent risk factor for prognosis according to the existing studies ( Supplementary Table S2 ). Furthermore, the relationship between clinical factors and CMTM6/CMTM4 expression was analyzed by Chi-squared test. Results showed that there is a significant association between CMTM6 expression and Ki67 expression/recurrence, while CMTM4 expression is not significantly different between patients with clinically-related factors ( Supplementary Table S3), indicating that CMTM6 may play an important role in tumor genesis and development.

Tumor microenvironment plays an important role in tumor progression, immune escape and drug resistance [7]. Why do patients with the same pathological type of tumor respond significantly differently to anti-PD-L1? This is largely due to differences in tumor microenvironments among patients with same tumors. Thus, it is imperative to optimize the existing immunotherapy strategies. TAMs are the predominant immune cell type expressing PD-L1, which play the regulatory role when these cells function as peptide-presenting cells [8]. This may be the key to the question why some patients with significant PD-L1 expression on tumor cells do not respond to PD-L1 blockade. Interestingly, we found that CMTM6/CMTM4 and PD-L1 co-localized in macrophages are associated with lower OS in glioma, suggesting that CMTM6/CMTM4 may be specific companion diagnostic biomarkers for immunotherapy in gliomas.

CMTM proteins control critical biological processes in cancer development and immune evasion [9]. For example, it has been proven that CMTM3, CMTM4, CMTM5 and CMTM7 are new potential tumor suppressors, and interestingly, patients with high CMTM6 expression have a worse survival prognosis than those with low CMTM6 expression according to this study. Therefore, CMTM6 may be involved in tumorigenesis in gliomas. It is reasonable to infer that the effect of CMTM6/CMTM4 in glioma patients may depend on the balance of its function in both tumor and the immune system. These results suggest that CMTM family genes might be tumor-related genes which have opposite roles. The expression levels of CMTM family members in tumor and adjacent tissue suggest that CMTM family may have potential application value in clinical diagnosis, individualized treatment and prognostic analysis of tumor.

In summary, we showed that CMTM6/CMTM4 expression is significantly correlated with PD-L1 in gliomas, which corresponds to the role of CMTM6/CMTM4 in the stabilization of PD-L1 in tumor cells. What is more, we found that macrophages are major cells expressing CMTM6/CMTM4 and PD-L1 in gliomas, which are associated with lower OS. This study suggests that CMTM6/CMTM4 may be specific companion diagnostic biomarkers for further guiding the immunological intervention in gliomas.

## Supplementary Data

Supplementary data is available at *Acta Biochimica et Biophysica Sinica* online.
